# Does early intensive multifactorial treatment reduce total cardiovascular burden in individuals with screen-detected diabetes? Findings from the ADDITION-Europe cluster-randomized trial

**DOI:** 10.1111/j.1464-5491.2012.03759.x

**Published:** 2012-11

**Authors:** R K Simmons, S J Sharp, A Sandbæk, K Borch-Johnsen, M J Davies, K Khunti, T Lauritzen, G E H M Rutten, M van den Donk, N J Wareham, S J Griffin

**Affiliations:** 1 MRC Epidemiology UnitCambridge, UK; 2 School of Public Health, Department of General Practice, University of ÅrhusÅrhus; 3 Institute of Public Health, Research Center for Quality in Health Care, University of Southern DenmarkOdense, Denmark; 4 Department of Cardiovascular SciencesLeicester, UK; 5 Department of Health Sciences, University of LeicesterLeicester, UK; 6 Julius Center for Health Sciences and Primary Care, University Medical Center UtrechtUtrecht, the Netherlands

## Abstract

**Aims:**

To describe the total cardiovascular burden (cardiovascular morbidity or mortality, revascularization or non-traumatic amputation) in individuals with screen-detected diabetes in the ADDITION-Europe trial and to quantify the impact of the intervention on multiple cardiovascular events over 5 years.

**Methods:**

In a pragmatic, cluster-randomized, parallel-group trial in four centres (Denmark; Cambridge, UK; the Netherlands; and Leicester, UK), 343 general practices were randomized to screening plus routine care (*n* = 1379 patients), or screening and promotion of target-driven, intensive treatment of multiple risk factors (*n* = 1678). We estimated the effect of the intervention on multiple cardiovascular events after diagnosis of diabetes using the Wei, Lin and Weissfeld method.

**Results:**

Over 5.3 years, 167 individuals had exactly one cardiovascular event, 53 exactly two events, and 18 three or more events. The incidence rates (95% CI) of first events and any event per 1000 person-years were 14.6 (12.8–16.6) and 20.4 (18.2–22.6), respectively. There were non-significant reductions in the risk of a first (hazard ratio 0.83, 95% CI 0.65–1.05) and second primary endpoint (hazard ratio 0.70, 95% CI 0.43–1.12). The overall average hazard ratio for any event was 0.77 (95% CI 0.58–1.02).

**Conclusions:**

Early intensive multifactorial treatment was not associated with a significant reduction in total cardiovascular burden at 5 years. Focusing on first events in cardiovascular disease prevention trials underestimates the total cardiovascular burden to patients and the health service.

## Introduction

Type 2 diabetes is a common chronic condition associated with a substantial burden of cardiovascular disease. In patients with established diabetes, risk of cardiovascular events and mortality can be reduced by treatment of individual risk factors [1–3] and by intensive multifactorial treatment [4]. More recently, research has focused on early detection and treatment of diabetes. The condition meets many of the criteria for screening [5] and there is growing evidence for the benefit of intensive treatment early in the course of the disease [6]. Population screening has been recommended by several national organizations and the National Health Service (NHS) includes assessment of diabetes in its Health Checks programme [7]. Consequently, more individuals will be found earlier in the disease trajectory, contributing to increased healthcare resource use. There are very few data on the cardiovascular experience of individuals following screen detection of diabetes and there is little current evidence for treatment recommendations in this high-risk group.

Results from ADDITION-Europe, a cluster-randomized trial of intensive, target-driven management of screen-detected patients, showed that, compared with routine care, intensive treatment was associated with a modest but statistically significant increase in treatment of multiple risk factors and a non-significant 17% relative reduction in the hazard of a first composite cardiovascular endpoint [8]. Examination of events in ADDITION-Europe demonstrated that a significant number of participants experienced more than one cardiovascular event. There is growing recognition that it is important to examine the total number of events during trial follow-up to appropriately reflect the effects of interventions on the burden of disease to patients and the health service [9–13]. This is particularly pertinent for chronic conditions, such as Type 2 diabetes, where trial follow-up is normally over several years to allow sufficient cardiovascular endpoints to accrue, and where individuals may experience multiple events. Further, recent improvements in the treatment of cardiovascular disease have changed the scope of relevant outcomes that prevention trials seek to capture. While cardiovascular disease event rates have reduced in the last 40 years, preventive interventions such as coronary angioplasty are on the increase [14]. Reporting only a ‘composite cardiovascular disease event’ endpoint may therefore mask a treatment effect; for example, the intervention might reduce the risk of one event but increase the risk of another [15].

We aimed to describe the total cardiovascular disease burden in ADDITION-Europe and quantify the impact of the intervention on multiple cardiovascular disease events. Furthering our understanding of the cardiovascular experience of individuals with screen-detected Type 2 diabetes will allow improvement in diabetes care and planning and targeting of healthcare resources.

## Patients and methods

### Study design

The study design and results for the primary and key secondary outcomes in the ADDITION-Europe trial have been reported [8,16–19]. In brief, ADDITION-Europe consists of a screening phase and a pragmatic, cluster-randomized parallel-group trial in four centres (Denmark, Cambridge UK, the Netherlands and Leicester UK). Of 1312 general practices invited to participate, 379 (29%) agreed and 343 clusters (26%) were independently randomized to screening plus routine care of diabetes, or screening followed by intensive multifactorial treatment. Allocation was concealed from patients throughout the trial. Population-based stepwise screening programmes among people aged 40–69 years (50–69 years in the Netherlands), without known diabetes, were undertaken between April 2001 and December 2006 [18–22]. Individuals were diagnosed with diabetes according to World Health Organization criteria [23]. General practitioners assessed patients against exclusion criteria: an illness with a life expectancy of less than 12 months, housebound, pregnancy or lactation, or psychological or psychiatric problems that were likely to invalidate informed consent. Overall, 3057 eligible participants with screen-detected diabetes agreed to take part (Denmark: 1533; Cambridge, UK: 867; the Netherlands: 498; and Leicester, UK: 159). The study was approved by local ethics committees in each centre. All participants provided informed consent.

### Intervention

The characteristics of the interventions to promote intensive treatment in each centre have been described previously [16–19]. Further details are available at the study website (http://www.addition.au.dk/). We aimed to educate and support general practitioners, practice nurses and participants in target-driven management (using medication and promotion of healthy lifestyles) of hyperglycaemia, blood pressure and cholesterol, based on the stepwise regimen used in the Steno-2 study [24]. Treatment targets and algorithms were common for the intensive treatment group in all centres and were based on trial data demonstrating the benefits of intensive treatment of cardiovascular risk factors in people with Type 2 diabetes [1,2,24–26]. Targets included HbA_1c_ < 53 mmol/mol (7.0%), blood pressure ≤ 135/85 mmHg, cholesterol < 5 mmol/l without ischaemic heart disease or < 4.5 mmol/l with ischaemic heart disease, and prescription of aspirin to those treated with anti-hypertensive medication. Following publication of the Heart Protection Study [27], the treatment algorithm included a recommendation to prescribe a statin to all patients with a cholesterol level ≥ 3.5 mmol/l. In the routine care group, patients received the standard pattern of diabetes care according to the current recommendations applicable in each centre [28–31].

### Measurement and endpoints

Health assessments at baseline and after 5 years included biochemical, anthropometric and questionnaire measures, and were undertaken by centrally trained staff following standard operating procedures and who were unaware of study group allocation. All biochemical measures were analysed in five regional laboratories at baseline and follow-up. Standardized self-report questionnaires were used to collect information on socio-demographic characteristics (education, employment and ethnicity), lifestyle habits (smoking status and alcohol consumption) and prescribed medication. Changes in biochemical measures and medication from baseline to 5-year follow-up have been reported previously [8].

Individuals were followed for a mean of 5.3 years. The primary outcome was time to first cardiovascular event after diagnosis of diabetes, including cardiovascular mortality, cardiovascular morbidity (non-fatal myocardial infarction and non-fatal stroke), revascularization and non-traumatic amputation. The revascularization endpoint included strictly defined invasive cardiovascular procedures (coronary artery bypass graft surgery, percutaneous coronary interventions and percutaneous coronary interventions attempt) and peripheral vascular procedures. See the study website for the endpoint manual (http://www.addition.au.dk/).

In each centre, participants’ medical records or national registers were searched for potential endpoints by staff unaware of group allocation. In Denmark, the national patient register was searched for deaths and for International Classification of Diseases (ICD)-10 codes for cardiovascular events and surgical procedures concerning amputations and revascularizations. In Cambridge and Leicester, participants were registered with the England and Wales Office of National Statistics, which provided copies of death certificates. Sensitive electronic searches of general practice records were conducted. If a possible event was highlighted, copies were made of medical records. Additional information was obtained from hospital records and coroner’s offices as required. In the Netherlands, investigators extracted endpoint and vital status information from general practice records onto standardized forms. All events for the primary outcomes analysis and for the total cardiovascular disease events outcome analysed in this paper were independently adjudicated by two members of a local endpoint steering committee, unaware of group allocation according to an agreed protocol using standardized case report forms.

### Statistical analysis

We summarized baseline characteristics of participants by the number of cardiovascular disease events that they experienced during the course of the ADDITION-Europe trial (after diagnosis of diabetes): no events, single event or multiple events. We then used the Wei, Lin and Weissfeld method to analyse multiple event data based on a stratified Cox proportional hazards model [32]. This method was originally proposed for the analysis of multivariate failure time data, but its application to recurrent events data such as the data from the ADDITION-Europe trial has also been justified [33]. The parameters (hazard ratios) of a standard Cox model are estimated by defining a separate ‘risk set’ each time an individual experiences an event, which includes all individuals who are at risk at the time of that particular event. Once an individual has experienced the event of interest, they are no longer at risk for subsequent events. The Wei, Lin and Weissfeld method adapts the standard Cox model by creating a separate stratum for each event (first event, second event, third event, etc.). Within the ‘second event’ stratum, for example, the risk set at each event time includes all individuals still in follow-up, regardless of whether or not they have already experienced a first event. Because each individual contributes to more than one stratum in the analysis, robust standard errors are calculated to take into account the lack of independence between contributions from the same individual. The Wei, Lin and Weissfeld method allows the estimation of separate effects of the intervention on the hazard of the first event, second event, third event, etc., based on groups comparable at the point of randomization; because randomization is preserved in the estimation of the intervention effects for events beyond the first event, these estimates are valid. The interpretation of the event-specific intervention effects is from the viewpoint of intervention onset.

Within each country, we estimated the effects of the intervention on the hazard of experiencing one, two, three and four events using hazard ratios and 95% confidence intervals. Consistent with the approach used previously [8], the country-specific log hazard ratios and standard errors were then combined using fixed-effects meta-analysis, and the *I*^2^-statistic, representing the proportion of variability (in log hazard ratios) between centres attributable to heterogeneity was calculated. The Wei, Lin and Weissfeld method involves the calculation of robust standard errors to allow for the fact that the same individual may contribute to more than one stratum of the model; it was not possible within Stata to also account for the fact that the individuals were clustered within general practices, as previously. However, the intracluster correlation coefficient for the primary endpoint was very small in all three countries (Denmark: 0.014; UK: 0.0000016; the Netherlands: 0.025) [8], and the hazard ratios and 95% confidence intervals for the primary endpoint changed minimally when practice-level clustering was ignored. In order to exclude the potential impact of centre-specific variations in healthcare practice, for example referrals for revascularization, we conducted a sensitivity analysis using a composite endpoint restricted to cardiovascular mortality, non-fatal myocardial infarction and non-fatal stroke. Type I error was set at 0.05 for all tests. All data were analysed using Stata version 11.0 (Stata Corp., Colllege Station, TX, USA).

## Results

Primary endpoint data were available for 3055/3057 (99.9%) participants. Compared with individuals who did not experience a cardiovascular disease event, individuals with one or more events were more likely to have had a previous myocardial infarction or stroke at baseline, to be older, male and a current smoker, and less likely to be employed (Table 1). Individuals with two or more events had lower values for weight, waist circumference and BMI at baseline, and higher values for HbA_1c_, total cholesterol and LDL cholesterol at baseline, compared with those with no or one event. There was no clear trend for blood pressure across the groups. There was a very clear trend of increased prescription of cardio-protective drugs across the groups; individuals who experienced two or more events during the trial were prescribed the highest rate of anti-hypertensive medication, cholesterol-lowering drugs and aspirin at baseline.

**Table 1:** Baseline characteristics in individuals with no events, single event or multiple events in the ADDITION-Europe trial [values are shown as mean (sd) unless specified]

Characteristics	No events (*n* = 2817)	Single event (*n* = 167)	Multiple events (*n* = 71)
Demographic variables			
Male gender, *n*%	1590 (56.4)	127 (76.1)	52 (73.2)
Age at diagnosis (years)	60.0 (6.9)	62.5 (6.4)	63.4 (6.1)
White ethnicity, *n*%*	2557 (94.1)	165 (98.8)	61 (91.0)
Employed, *n*%*	845 (41.8)	42 (31.3)	19 (34.5)
Clinical variables			
History of myocardial infarction, *n*%*	149 (5.6)	31 (19.9)	8 (13.1)
History of stroke, *n*%*	58 (2.2)	5 (3.3)	6 (9.8)
Current smokers, *n*%*	731 (26.5)	61 (37.4)	27 (39.1)
Median (IQR) units of alcohol/week	4 (1.0–13.0)	4 (1.0–12.0)	6 (1.0–14.0)
BMI (kg/m^2^)	31.6 (5.6)	32.0 (6.2)	30.4 (4.9)
Weight (kg)	90.6 (17.5)	92.5 (18.6)	86.9 (16.1)
Waist circumference (cm)	106.9 (13.5)	109.4 (14.0)	105.0 (12.2)
Median (IQR) IFCC HbA_1c_, mmol/mol	48 (43–56)	50 (45–58)	52 (44–65)
Median (IQR) DCCT HbA_1c_, %	6.5 (6.1–7.3)	6.7 (6.3–7.5)	6.9 (6.2–8.1)
Systolic blood pressure (mmHg)	148.8 (21.7)	152.2 (22.2)	150.9 (23.4)
Diastolic blood pressure (mmHg)	86.3 (11.1)	86.1 (12.7)	84.8 (11.0)
Total cholesterol (mmol/l)	5.6 (1.1)	5.4 (1.2)	5.9 (1.6)
Median (IQR) HDL cholesterol (mmol/l)	1.2 (1.0–1.5)	1.1 (1.0–1.4)	1.2 (1.0–1.5)
LDL cholesterol (mmol/l)	3.4 (1.0)	3.4 (1.0)	3.6 (1.3)
Median (IQR) triglycerides (mmol/l)	1.7 (1.2–2.4)	1.6 (1.2–2.5)	1.6 (1.1–2.8)
Self-reported medication, *n* (%)			
Any anti-hypertensive drugs	1,212 (44.7)	84 (51.9)	39 (54.9)
Any cholesterol-lowering drugs	414 (15.3)	42 (25.9)	23 (32.4)
Aspirin	350 (12.9)	46 (28.4)	22 (31.0)

* Numbers may not add up to total because of missing values.

DCCT, Diabetes Control and Complications Trial; IFCC, International Federation of Clinical Chemistry; IQR, interquartile range; sd, standard deviation.

Of the 3055 individuals with endpoint data in the ADDITION-Europe trial, 238 experienced a first cardiovascular disease event, 71 experienced a second event and 25 experienced three or more events (Table 2). The incidence rates (95% CI) of first events and any event per 1000 person-years were 14.6 (12.8–16.6) and 20.4 (18.2–22.6), respectively. The most frequent first events were revascularizations (37%), non-fatal myocardial infarction (26%) and cardiovascular disease death (20%). The corresponding percentages for the second events were 77, 7 and 11%, respectively. Fifty-two per cent (33/63) of individuals experienced a revascularization within 30 days of any myocardial infarction. The number of individuals experiencing three or more events was too small to enable comparison of categories of events. Only two amputations were reported, one in each trial group, and not for the first events.

**Table 2:** Type of cardiovascular event by first, second and third or more events in the ADDITION-Europe trial (values are number of individuals)

	Routine care (*n* = 1377)	Intensive treatment (*n* = 1678)	Total (*n* = 3055)
First event (*n* = 238)			
Cardiovascular disease death	22 (19%)	26 (21%)	48
Myocardial infarction	32 (27%)	29 (24%)	61
Stroke	19 (16%)	22 (18%)	41
Revascularization	44 (38%)	44 (36%)	88
Amputation	0	0	0
Second event (*n* = 71)			
Cardiovascular disease death	3 (8%)	5 (15%)	8
Myocardial infarction	5 (13%)	0	5
Stroke	1 (3%)	1 (3%)	2
Revascularization	28 (74%)	27 (82%)	55
Amputation	1 (3%)	0	1
Three or more events (*n* = 25)			
Cardiovascular disease death	3	1	4
Myocardial infarction	3	0	3
Stroke	0	2	2
Revascularization	11	4	15
Amputation	0	1	1

**FIGURE 1:**
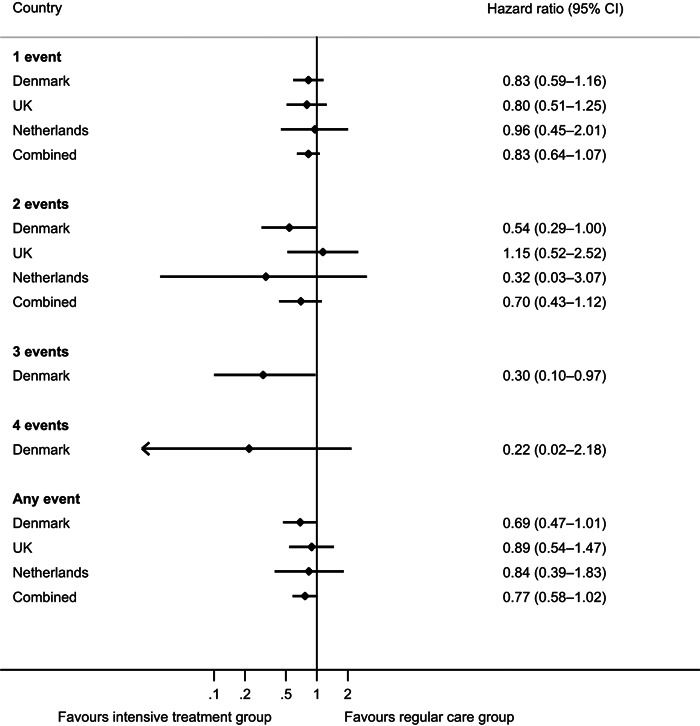
The effect of the intervention on the hazard of experiencing one, two, three, four and any event. Each intervention effect is presented as a hazard ratio within each country and combined across countries using fixed-effects meta-analysis. Only Denmark had sufficient numbers of individuals to estimate an intervention effect for three and four events. All *I*^2^-values for each comparison were 0%.

As shown previously [8], the hazard ratio for a first event comparing intensive treatment with routine care was 0.83 (95% CI 0.64–1.07) (*I*^2^ = 0%) (Fig. 1). The hazard ratio for a second event was 0.70 (95% CI 0.43–1.12) (*I*^2^ = 0%). Only Denmark had sufficient numbers of individuals to estimate a hazard ratio for a third and fourth event. The overall average hazard ratio for any event was 0.77 (95% CI 0.58–1.02) (*I*^2^ = 0%). When replicating the analysis using a cardiovascular endpoint restricted to cardiovascular mortality, non-fatal myocardial infarction and non-fatal stroke, the hazard ratio for a first event comparing intensive treatment with routine care was 0.86 (95% CI 0.63–1.16) (*I*^2^ = 0%).

## Discussion

An intervention to promote target-driven, intensive management of individuals with screen-detected diabetes was not associated with a significant reduction in total cardiovascular burden at 5 years. Differences between study groups for the first, second, third and fourth events, and for the overall average hazard, favoured the intensive treatment group, and the point estimates for these events were lower than for the first event; however, we cannot rule out the possibility that these findings were attributable to chance. Results from this analysis provide a novel insight into the cardiovascular experience of individuals with screen-detected Type 2 diabetes in the 5 years following diagnosis. Recurrent cardiovascular disease incidence rates are lower than those observed in cohorts of individuals with long-standing diabetes [34], and there was a lower than expected incidence of first cardiovascular events (8.5% in the routine care group). The trial was undertaken during a period of improvement in diabetes care in general practice, such as that associated with the introduction of the Quality and Outcomes Framework for primary care in the UK [35], and evidence-based guidelines in Denmark [36] and the Netherlands [37], reducing the achievable differences in treatment between groups. Further, adherence to treatment algorithms may have been suboptimal in this pragmatic trial, which may have contributed to the modest differences in treatment between groups. It remains to be seen whether intensification of early treatment in screen-detected individuals might translate into improved outcomes in the longer term.

Given the significant number of individuals with more than one cardiovascular event, focusing on first events in cardiovascular prevention trials may underestimate the total cardiovascular burden to patients and the health service. Our results also demonstrate that subsequent events are often dependent on the first event; there was a difference in the frequency of types of first and second events in ADDITION-Europe. The most frequent first cardiovascular disease events were revascularization (37%) and non-fatal myocardial infarction (26%), which corresponds to the most frequent events in the Steno-2 study [4] (revascularization 28% and myocardial infarction 21%). However, a very high proportion of the second events were also revascularizations (77%). This finding suggests some dependence between the first and second event, given that many of the revascularizations (52%) followed an acute myocardial infarction, and many within a relatively short time period. This suggests that we should continue to focus on first events to generate estimates of efficacy on composite outcomes in trials. After excluding revascularizations and amputations from the composite endpoint, the overall results comparing the rate of first event between the treatment groups remained the same. There was no evidence that multifactorial treatment was associated with harmful effects in participants who have experienced a cardiovascular disease event in the first few years after detection of diabetes by screening.

### Strengths and limitations

Participants were drawn from large and representative population-based samples in three different European countries. There was a high level of participant retention. Endpoints were strictly defined and independently adjudicated in both trial groups. As practices in the same regions, served by the same hospitals, were randomized, the underlying referral criteria and hospital care would not have been differential by study group. The Wei, Lin and Weissfeld method allowed analysis of multiple cardiovascular disease events, which better reflects the impact of the intervention on the total burden of cardiovascular disease among screen-detected patients. The generalizability of our findings should be interpreted with caution given the non-random recruitment of general practices, notwithstanding the large geographical area covered in each country, and the nationally representative characteristics of the 26% of invited practices that took part [18,19,21]. The 5-year duration of follow-up may have been insufficient given the lower than expected event rate, and suggests that follow up of ADDITION-Europe may be justified to examine whether early intensive treatment reduces cardiovascular risk in the longer term, as seen in the UK Prospective Diabetes Study (UKPDS) [38]. The Wei, Lin and Weissfeld method does not take into account how one event may affect the risk of a subsequent event [12]. This is particularly pertinent for the pattern of events we observed in the ADDITION-Europe trial where there appeared to be a dependency between certain types of events. In particular, the overall average hazard ratio should be interpreted with caution as it assumes that the effect of the intervention on the hazard of a first event, second event, etc. is the same [33]. The small number of individuals experiencing third and fourth events made it difficult to draw any conclusions about potential risk reduction in these groups.

Exploration of multiple events has been carried out in other cardiovascular disease prevention trials examining single therapies in individuals with acute coronary syndrome [9,10], previous myocardial infarction [12] and clinically evident coronary heart disease [11]. Recurrent events are associated with increased testing, hospital visits, medications, practitioner time and costs, and are likely to result in higher levels of morbidity and decreased quality of life [11]. Further, different events may have different impacts on health costs and quality of life [15]. Examining multiple events during the course of cardiovascular disease prevention trials might therefore warrant consideration in future trials given the implications for healthcare resource utilization [10]. We are undertaking analyses of trial data on health service costs and plan to incorporate total number of cardiovascular disease events to provide a more comprehensive estimate of total cost-effectiveness.

## Conclusion

Early intensive multifactorial treatment was not associated with a significant reduction in total cardiovascular burden at 5 years. Focusing on first events in cardiovascular disease prevention trials underestimates the total cardiovascular burden to patients and the health service.
